# Comparison of Treatment Modalities for Locally Advanced Gastric Cancer: A Propensity Score Matching Analysis

**DOI:** 10.7150/jca.41082

**Published:** 2020-05-18

**Authors:** Jianglong Han, Zhihua Nie, Ping Li, Hongwei Shi, Shijie Wang, Qin Li, Rui Zhang, Yunfeng Qiao, Kejie Huang, Zhenming Fu

**Affiliations:** 1 Cancer Center, Renmin Hospital of Wuhan University, Wuhan, 430060, China; 2 Department of Radiation and Medical Oncology, Zhongnan Hospital of Wuhan University, Wuhan, 430071, China; 3 Taikang Tongji (Wuhan) Hospital, Wuhan, 430050, China

**Keywords:** Gastric cancer, D1 dissection, D2 dissection, Adjuvant therapy, Propensity score matching, SEER

## Abstract

**Background**: A consensus regarding optimum treatment strategies for locally advanced gastric cancer (LAGC) has not yet been reached. We aimed to evaluate the efficacy of various treatment modalities for LAGC and provided clinicians salvage options under real-world situation.

**Methods**: Medical charts of patients with LAGC who underwent radical resection plus adjuvant chemotherapy or chemoradiotherapy from July 2003 to December 2014 were included. Validation cohort were selected from SEER database between 2004 and 2014. Kaplan-Meier and Cox proportional hazardous models were used to evaluate the overall survival (OS), cancer-specific survival (CSS), and disease-free survival (DFS). Propensity score matching (PSM) was used to adjust for potential baseline confounding.

**Results**: A total of 350 patients were included and divided into D1 dissection plus chemotherapy group (D1CT, n = 74), D1 dissection plus adjuvant chemoradiotherapy group (D1CRT, n = 69), D2 dissection plus adjuvant chemotherapy group (D2CT, n = 134), and D2 dissection plus adjuvant chemoradiotherapy group (D2CRT, n = 73). PSM identified 50 patients in each group. After PSM, better DFS (*P* for D2CRT vs. D1CT, D1CRT, and D2CT was 0.001, 0.006, and 0.001, respectively) and OS (*P* for D2CRT vs. D1CT, D1CRT, and D2CT was 0.001, 0.011, and 0.022, respectively) were found for the D2CRT group (mean, OS = 110.7months, DFS = 95.2 months) than the other groups. Similar findings were further validated in the Surveillance, Epidemiology, and End Results database (SEER) cohort. In addition, patients in the D1CRT group achieved similar survival outcomes to those in the D2CT group (mean OS, 72.8 vs. 59.1 months, *P* = 0.86; mean DFS, 54.4 vs. 34.1 months, *P* = 0.460).

**Conclusions**: The results of the study indicated the better role for D2CRT in treating the LAGC, meanwhile, the patients treated with D1CRT might achieve similar survival as that of D2CT patients.

## Introduction

Gastric cancer is the fifth most common malignancy and the third leading cause of cancer-related death worldwide 1. East Asia, including China, Japan, and Korea, has the highest incidence rates of gastric cancer. In 2014, 410,400 new stomach cancer cases and 293,800 cancer-related deaths were estimated to have occurred in China 2.

Adequate radical resection with adjuvant therapies may be the possible curative therapy for locally advanced gastric cancer (LAGC). However, the preferred treatment for LAGC differs by geographical region. The recommended adjuvant therapies are perioperative chemotherapy or postoperative chemoradiotherapy in the USA and some other parts of the world 3-5, whereas the postoperative chemotherapy is preferred in Japan and South Korea 6, 7. Clearly, there is no universally accepted optimal strategy for LAGC treatment.

For lymph node dissection, D2 dissection is generally recommended because of its lower recurrence and cancer-related death rates than D1 dissection 8, 9. However, D2 dissection may be associated with higher postoperative mortality and morbidity 10. In addition, the more complicated surgical technique and prolonged operation time make D2 dissection most commonly practiced in high-volume centers with experienced surgeons 9, 11, 12. In developing areas such as rural China, D2 dissection cannot be performed universally or is practiced in a nonstandard way due to inadequate surgical resources 13. In Western countries, D1 or even D0 resections are still often performed for various reasons 14, 15. Under these circumstances, salvage treatments should be sought after an inadequate lymphadenectomy. The advancement in adjuvant therapies may provide opportunities to compensate, at least partially, for the defects of imperfect lymphadenectomy. Currently, various therapeutic strategies are used in the treatment of LAGC, including but not limited to D1 dissection plus adjuvant chemotherapy (D1CT), D1 dissection plus adjuvant chemoradiotherapy (D1CRT), D2 dissection plus adjuvant chemotherapy (D2CT), and D2 dissection plus adjuvant chemoradiotherapy (D2CRT).

It is recommended in China that D2 dissection should be pursued in the treatment of LAGC 16; thus, a randomized controlled trial (RCT) containing multiple control arms of other resection modalities might be inappropriate. Therefore, we compared the efficacy among various therapeutic strategies which were currently used in the treatment of LAGC in a retrospective cohort, through conventional and propensity score matching (PSM) approaches.

## Participants and Methods

### Participants

The study was performed at an affiliated Hospital of Wuhan University (Hubei, China), the Institutional Review Board approval was not required according to the institution policy because the study is a retrospective medical chart review without direct patient contact. Additional individual consents for this analysis was not needed. Medical records of newly diagnosed LAGC patients with radical operation plus adjuvant therapies in the affiliated hospital between July 2003 and December 2014 were collected. Eligible patients had to fulfill the following criteria: age between 20 years old and 75 years old, histologically confirmed gastric adenocarcinoma, R0 gastrectomy with D2 (en bloc with the N1 and N2 lymph nodes and a minimum of 15 lymph nodes examined) or D1 dissection, no evidence of gross peritoneal seeding and metastasis, stage ⅠB-ⅢC (as defined by the American Joint Committee on Cancer staging manual, 7th edition) 17, and having received at least 2 cycles of either adjuvant or concurrent chemotherapy. All patients had adequate hematologic, hepatic, and renal function tests. Excluded from the study were patients who were: T1N0M0 disease; Siewert type I/II or gastro-esophageal junction disease; no radical gastrectomy; non-R0 resection, and with distant metastases. Pretreatment evaluations consisted of medical history assessment, physical examination, hematologic and biochemical analyses, tumor marker evaluations, electrocardiography, and computed tomography (CT) of the thorax, abdomen (contrast enhanced), and pelvis. As a result, a cohort of 350 cases was included in the current study. According to the types of treatment modalities, patients were categorized into four groups, i.e., D1CT group, D1CRT group, D2CT group, and D2CRT group (74 cases, 69 cases, 134 cases, and 73 cases, respectively).

**Figure 1:**
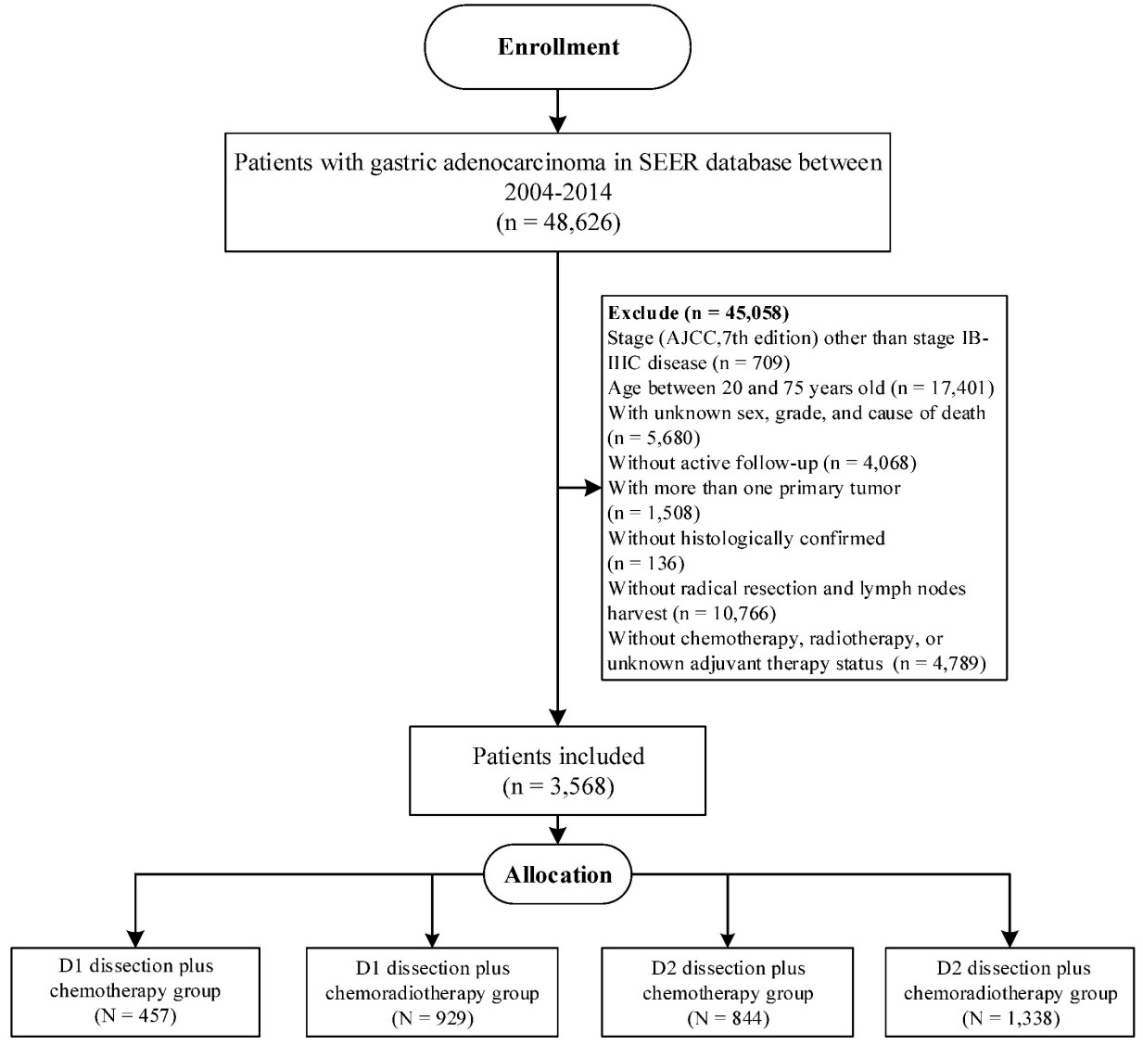
The flowchart of SEER validation group selection.

Furthermore, we aimed to validate our findings in Surveillance, Epidemiology, and End Results (SEER) database, which covered approximately 34.6% of the US population 18. Eligible participants were included according to the inclusion criteria in our study (**Figure 1**), and the selected patients were categorized into D1CT group, D1CRT group, D2CT group, and D2CRT group (457 cases, 929 cases, 844 cases, and 1,338 cases, respectively).

### Treatment

All patients underwent radical resection with lymph node dissection. In this study, radiotherapy regimens were conformal radiotherapy or intensity-modulated radiotherapy (IMRT). All patients were treated following a standard postoperative CCRT protocol which has been previously described in a phase II trial 19. For postoperative chemotherapy, the standard regimen for most patients was FOLFOX4 (85 mg/m^2^ oxaliplatin intravenous [IV] infusion on day 1; 2 hours IV infusion of 200 mg/m^2^ leucovorin [LV] on days 1-2; 400 mg/m^2^ 5-fluorouracil [5-FU] IV bolus on days 1-2, followed by 22-hour continuous IV infusion of 600 mg/m^2^ 5-FU on days 1-2 every two weeks). Patients in the concurrent chemoradiation group received two cycles of FOLFOX4, subsequently, they received radiotherapy (45 Gy at 1.8 Gy per fraction, 5 fractions per week for 5 weeks) with a 5-FU/LV regimen (400 mg/m^2^ 5-FU IV bolus + 20 mg/m^2^ LV IV bolus per day on the first 4 and the last 3 days of radiotherapy), followed by additional cycles of FOLFOX4. However, in this study, mFOLFOX6, CAPOX, FOLFORI, and 5-FU IV plus another chemotherapeutic drug (such as mitomycin C, cisplatin) were also allowed for chemotherapy. Tegafur, capecitabine, or 5-FU IV regimen was also allowed for concurrent chemoradiotherapy. (**Table S1**)

### Follow-up and toxicity

Follow-up after completion of treatment consisted of visits every three months for the first two years, then every six months for the following three years, and annually thereafter. Documented disease progression was defined according to physical examination and/or imaging or biopsy confirmation. New lymph nodes measuring over 1 cm on CT or MRI in the short axis diameter were considered malignant. Acute toxicities, measured from the initiation of treatment to 90 days after completion, were graded using the National Cancer Institute's Common Terminology Criteria for Adverse Events version 3.0 20. Adverse effects were categorized as hematologic adverse events, gastrointestinal adverse events, liver toxicity, and other toxicity in this study.

### Statistical analysis

PSM is a tool to reduce selection bias in nonrandomized studies 21. Propensity score with nearest neighbor matching was performed in our study to reduce the selection bias and ensure baseline balance among treatment groups, using the calipers equal to 0.05 of the standard deviation of the logit of the propensity score. Standardized differences were computed for every two groups, and the median standardized difference was utilized to show whether the distribution among groups reached balance 22. The covariates selected for matching were based on prior literature reports, known clinically prognostic factors, and availability in medical records. Selected variables included age, gender, tumor location, differentiated degree, metastatic lymph node ratio (MLR), and stage for primary cohort 23-25. The propensity scores were generated stepwisely 26. Variables were selected through univariate and then multivariable Cox regression models. Variables remained significant in the final model were selected to generate propensity scores. The distribution among groups was considered well-balanced if the standardized difference of variables < 10% 22.

The primary endpoints were overall survival (OS) and disease-free survival (DFS) for primary cohort; the endpoints were OS and cancer-specific survival (CSS) for validation cohort. Treatment-related toxicity was the secondary endpoint for primary cohort. OS was defined as the time from start of treatment to death from any cause; DFS was calculated from the date of surgery to the date of first disease progression (locoregional recurrence or metastasis). Locoregional recurrence was defined as any relapse at the remnant stomach, anastomosis site, tumor bed, or regional lymph nodes; CSS was defined as the time from treatment to death related to LAGC. Frequencies and proportions, as well as means were reported for categorical and continuous variables, respectively. General linear models or χ^2^-test were performed to compare the distribution of baseline characteristics. The Kaplan-Meier method was used for estimating survival curves. The Cox proportional hazardous model was performed to evaluate the hazard ratio (HR) and 95% confidence interval (CI) of the associations of survival with each clinical factor. *P ≤* 0.05 (2-sided probability) was considered statistically significant, *P ≤* 0.008 (2-sided probability) was considered statistically significant for pairwise comparison adjusted by Bonferroni method. All analyses were conducted using SAS 9.4 (SAS Institute, Cary, NC) and R software (Version 3.5.3).

## Results

### Baseline characteristics

**Figure 2:**
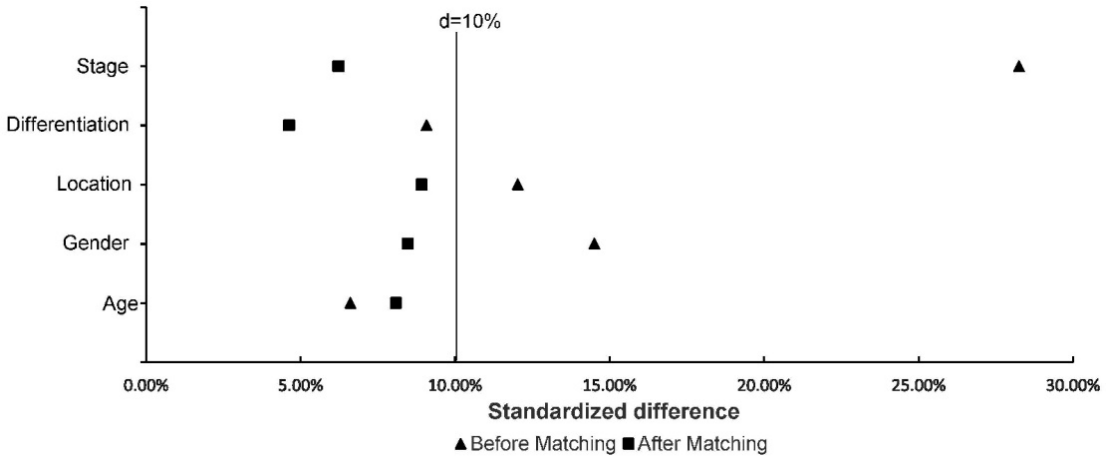
Standardized difference of study groups before and after matching. The distribution among groups was well balanced if the standardized difference < 10%.

**Table 1** and** Table 2** describe the baseline characteristics and survival outcomes before and after matching, respectively. From July 2003 to December 2014, a total of 350 patients treated with D1CT (n = 74), D1CRT (n = 69), D2CT (n = 134), and D2CRT (n = 73) were identified. After PSM, 50 patients in each group were left. The study was mainly carried out in males (> 60%), patients with stage III (> 60%) and poorly differentiated (> 70%) gastric adenocarcinoma. Slightly more elderly patients (56%) than young patients were included in the study.** Table 3** demonstrates the baseline characteristics for SEER cohort. In line with the primary cohort, old males (> 70%) with advanced stage (> 50%) and poorly differentiated adenocarcinoma (>70%) were included in validation group. Moreover, white patients accounts for 62.5% in the SEER cohort. **Figure 2** shows the standardized difference among primary cohort before and after PSM. Although age and differentiation degrees for all groups were similar and only changed slightly after PSM, the standardized difference was still less than 10%. The standardized difference of other variables was less than 10% after PSM, and all variables were balanced after PSM.

### Survival analysis

Since the median survival in many treatment groups has not yet been reached, we present the means of survival months. As shown in the **Table 1** and **Table 2**, 194 disease progression events occurred (52 in D1CT, 34 in D1CRT, 86 in D2CT, 22 in D2CRT groups, respectively) before matching, the lowest recurrence rate was observed in D2CRT group (30.1% in D1CRT vs. 64.2% in D2CT, 49.3% in D1CRT, and 70.3% in D1CT, *P* < 0.05 for D2CRT vs. D1CT, D1CRT, and D2CT, respectively). After matching, the recurrence rate remained lowest in the D2CRT group (32.0% vs. 64.0% in D2CT, 62.0% in D1CRT, and 66.0% in D1CT, respectively).

**Figure 3:**
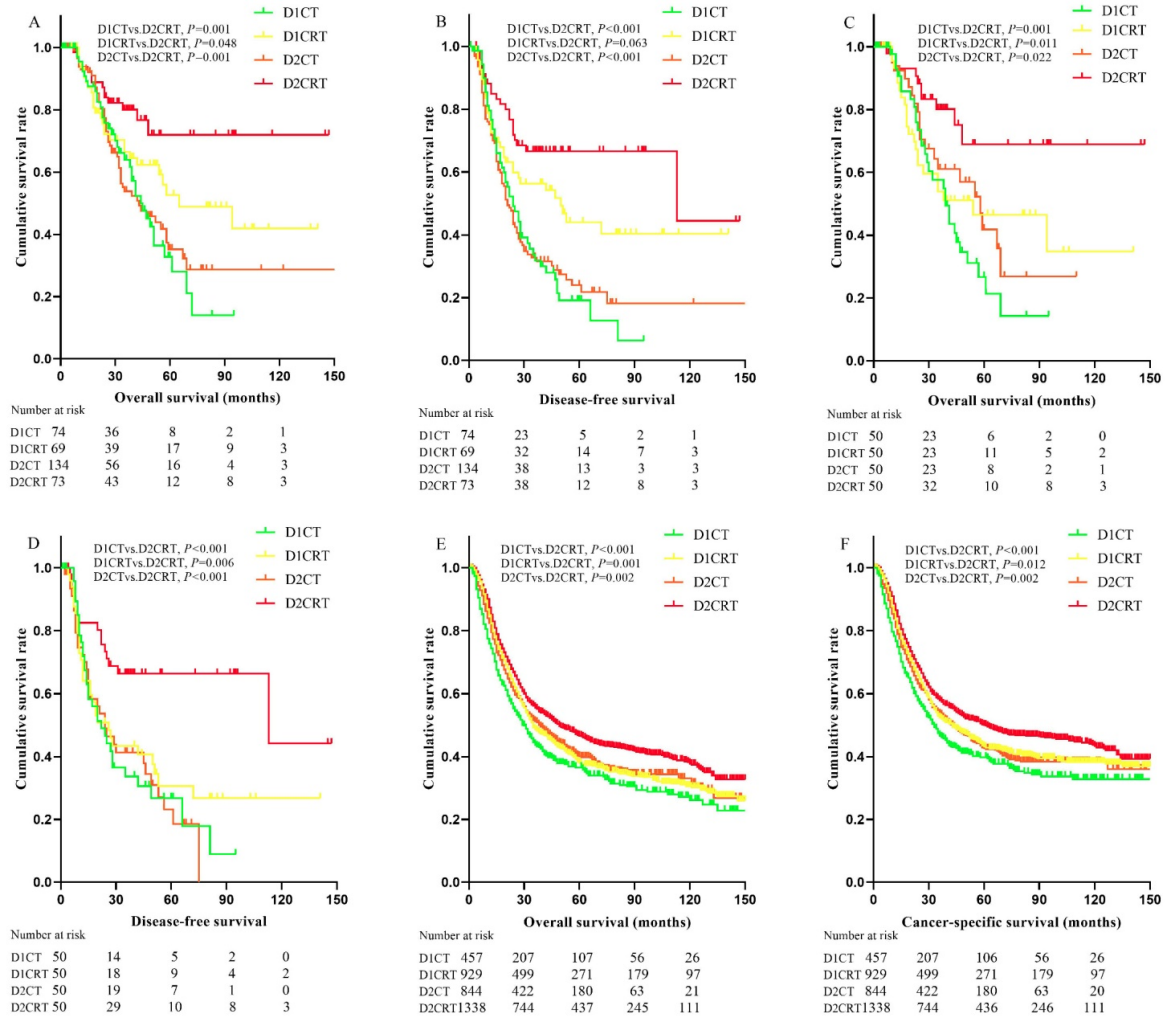
Kaplan-Meier curves of survival for study groups and SEER validation group. The Kaplan-Meier curves of OS (A) and DFS (B) for the study group before matching; the Kaplan-Meier curves of OS (C) and DFS (D) for the study group after matching; the Kaplan-Meier curves of OS (E) and CSS (F) in the SEER validation group. OS: overall survival, DFS: disease-free survival, CSS: cancer-specific survival, SEER: Surveillance, Epidemiology, and End Results program.

Better DFS were found in the D2CRT group compared with other groups both before matching (mean DFS for D1CT, D1CRT, D2CT, and D2CRT was 33.4 months, 71.3 months, 46.3 months, and 95.8 months, respectively; *P* for D2CRT vs. D1CT, D1CRT, and D2CT was 0.001, 0.063, and 0.001, respectively), similar results were also observed after matching (**Figure 3B, 3D**). The DFS between D1CRT and D2CRT groups became significantly different after matching (*P* = 0.006). Similar outcomes were found for OS among these groups before matching, however, the pairwise comparison among D1CRT, D2CT, and D2CRT in OS (mean OS for D2CRT, D1CRT, and D2CT was 110.7 months, 72.8months, and 59.1 months; *P* for D2CRT vs. D1CRT, and D2CT was 0.011, 0.022, respectively) showed no difference after matching (**Figure 3A, 3C**). Furthermore, similar results were observed in the validation cohort, highest OS (*P* for D2CRT vs. D1CT, D1CRT, and D2CT was <0.001, 0.001, and 0.002, respectively) and CSS (*P* for D2CRT vs. D1CT, D1CRT, and D2CT was <0.001, 0.012, and 0.002, respectively) were seen in the D2CRT group (**Figure 3E, 3F**). Multivariate analysis consistently indicated that D2CRT was a positive prognostic factor for both OS (HR and [95%CI] for D1CT, D1CRT, and D2CT vs. D2CRT was 3.3 [1.6-6.9], 2.4 [1.1-5.1], and 2.3 [1.1-5.0]) and DFS (HR and [95%CI] for D1CT, D1CRT, and D2CT vs. D2CRT was 2.7 [1.5-4.9], 2.1 [1.1-3.9], and 2.9 [1.6-5.3]), and the high MLR (*P* < 0.05) was associated with elevated risk of OS (HR = 1.5, 95%CI = 1.0-2.3) and CSS (HR = 1.63, 95%CI = 1.2-2.2) (**Table 4**). Moreover, the validation group also revealed the positive prognostic role of D2CRT and lower MLR for both OS and CSS (**Table S2** and**Figure S1**).

### Adverse effect analysis

**Table S3** describes the adverse events reported during the follow-up duration. Common adverse reactions occurred in the hematological system and gastrointestinal tract. Before matching, 187 adverse events were found. Grade 3 and 4 adverse events among the four groups showed no significant difference (*P* > 0.05 for every two comparison groups, data not shown). However, the incidence of grade 1 and 2 adverse events in D1CRT was significantly higher than that in the other three groups (*P* < 0.001 for every two comparisons). No statistical differences were found among the other three groups. After matching, similar outcomes were found for the occurrence of grade 3 and 4 adverse events. In contrast, D2CRT had statistically fewer grade 1 and 2 events than that of D1CRT (*P* = 0.021).

### Subgroup analysis

We performed a subgroup analysis according to the surgery time before and after 2011 (**Figure S2**). A total of 207 patients with D2 dissection (134 cases with D2CT and 73 cases with D2CRT) were enrolled; 84 cases of D2 dissection (40.6%) were performed before 2011. Overall, the highest 5-year OS and 5-year DFS were most observed in subgroups of D2CRT. Compared with D2CRT, D2CT had significantly reduced OS and DFS for patients who received surgery before 2011 both before and after matching. For patients who received operation after 2011, the DFS of the D2CT groups were significantly reduced compared with the D2CRT group before matching (D2CT vs. D2CRT, *P* = 0.001). However, the OS between D2CT and D2CRT groups did not show significant difference after matching (*P* = 0.11).

## Discussion

To the best of our knowledge, the present study was the first analysis to use PSM to compare current therapeutic modalities in the treatment of patients with newly diagnosed LAGC, and SEER cohort were further used to validate our findings. In this study, we found significantly better survival outcomes in the D2CRT group than the other treatment groups. In addition, we found that the D1CRT group showed no significant differences from the D2CT group in clinical outcomes, which might indicate that radiotherapy might compensate for the defects of imperfect lymphadenectomy to a large extend for patients with LAGC.

Adjuvant therapies are used to further improve the survival and quality of life in combination with surgery. Although strategies of adjuvant therapies vary among Eastern and Western countries, the survival benefits of adjuvant therapies after resection of LAGC are universally acknowledged 11. The Intergroup 0116 study showed that postoperative chemoradiotherapy improved both locoregional control rate and overall survival compared to surgery only 3. In our SEER cohort, which might partially represent the American population, survival benefit was also observed in chemoradiotherapy group. However, the trial has been criticized for the inadequate surgical procedures. In fact, whether postoperative chemotherapy or chemoradiotherapy should be the preferred choice after D2 dissection of LAGC was still under debate. In East Asia, the Korean ARTIST trials found no significant differences between postoperative chemoradiotherapy and postoperative chemotherapy after D2 dissection, but an improved DFS was found in node-positive patients 27, 28. Furthermore, the interim result of ARTIST 2 trial indicated that postoperative chemoradiotherapy achieved similar DFS to that of chemotherapy alone in the D2-resected node-positive LAGC patients 29. Meanwhile, a phrase Ⅲ trial in China reported a significantly improved DFS in the D2CRT group 30, and similar outcomes were also observed in several previous studies 31, 32. The European CRITICS trial 33 reported that postoperative chemoradiotherapy did not improve the survival outcome compared with postoperative chemotherapy in patients who received at least D1+ dissection. In our study, the results were partially consistent with the results of the study in China in terms of DFS 30. Furthermore, a benefit to OS was also found in our current study and in the SEER cohort. For radiotherapy, anterior-posterior opposed fields were utilized in the Korean ARTIST trials. In our study, most patients received IMRT as previously described which was dosimetrically superior to the conventional therapy used in ARTIST 19. This might partially explain the improved OS and DFS found in the current study. We found that D1CRT obtained similar OS and DFS as that of D2CT. Although little RCTs had directly compared the efficacy of D1CRT and D2CT, an article discussed the possibility for D1CRT to replace D2 dissection 30; it showed that adjuvant chemoradiotherapy was associated with better survival for patients with D1 dissection or R1 resection. Furthermore, 1:1 PSM among D1CT, D1CRT and D2CT groups were conducted to investigate the survival outcomes. We found that survival among D1CRT and D2CT remained similar. Furthermore, D1CRT in the validation group also showed a similar survival compared to D2CT (HR = 0.96, 95%CI = 0.88-1.14), whereas the elevated risk was observed when compared with D1CT group (HR = 1.19, 95%CI = 1.01-1.41). These findings highlight the beneficial effects of including postoperative radiotherapy in improving survival after surgery, especially in high incidence but resources-limited areas where D2 dissection is often performed in a nonstandard way.

Furthermore, we conducted subgroup analyses by the time of surgery before or after 2011, because the understanding and implementation of the standard D2 dissection did not reach a consensus till 2011 in the hospital where the study began. It was uncertain whether the quality of earlier D2 dissections was satisfactory. Before 2011, the D2CRT group had significantly improved 5-year OS and 5-year DFS rates compared with the D2CT group both before and after matching. For patients who received surgery after 2011, D2CRT was associated with a significantly improved DFS than D2CT, but no differences were observed for OS. Thus, our results support the possibility of improving DFS for postoperative chemoradiotherapy after standard D2 dissection. The findings indicated the efficacy of D2CRT in real-life situation. Moreover, our survival outcomes among the D2CT group (5-year OS survival, 76.1% vs. 73%) and the D2CRT group (5-year OS survival, 83.6% vs. 75%) after 2011 were comparable with those of the ARTIST trial. Therefore, although the cohort was highly selective, the quality of treatment of our patients was satisfactory. For adverse events, the number of grade 3 and 4 adverse events reported among the four groups was not significantly different or was even lower than those in other reports 33, 35. Furthermore, the rates for grade 1 and 2 adverse events (30-50%) were similar to those in reports from other Eastern countries 35, 36.

Our findings must be interpreted within the context of several limitations. Firstly, findings from the study were limited for the retrospective setting, but we recruited SEER cohort which might represent the American population to validate and extrapolate our findings. Moreover, it is inappropriate to design RCTs to compared the multiple treatment arms containing other suboptimal resection methods. Secondly, we selected the patients mainly based on completeness of clinical information. Most of the patients selected were residents of the local city and follow-ups were convenient to be performed. Patients in the study received major kinds of adjuvant treatments, these treatments might be heterogenous. However, this might be more representative of the real world to some extent. Thirdly, in the design of this study, patients who received neoadjuvant therapy were excluded since neoadjuvant therapy is not routinely performed in our institution. We found that most of the patients who received neoadjuvant therapy were first treated in local smaller hospitals before they received an operation opportunity in our hospital. This study is in no way intended to advocate for D1 dissection nor support D1CRT as an alternative to D2CT or D2CRT. Rather, we suggest that D2 dissection should be attempted whenever possible for LAGC, including in resource-limited areas. In fact, this study is intended to find practical options for oncologists under resource-limited conditions when D2 dissection is not optimally achieved.

In conclusion, we highlighted the beneficial effects of postoperative radiotherapy in current therapeutic modalities for treating LAGC. Given the lack of feasibility to design RCTs and compare the currently available modalities directly, although we suggest that D2 dissection should be attempted whenever possible, we recommend LAGC patients for postoperative radiation therapy when patients received suboptimal surgery.

## Supplementary Material

Supplementary figures and tables.

## Figures and Tables

**Table 1:** Baseline characteristics and survival outcomes of study groups before matching.

Characteristic	D1CT	D1CRT	D2CT	D2CRT	*P*^1^	*P*^1^	*P*^1^
	(n=74)	(n=69)	(n=134)	(n=73)	D1CT vs. D2CRT	D1CRT vs. D2CRT	D2CT vs. D2CRT
Age (years)	.				0.67	0.53	0.37
<55	32 (43.2%)	31 (44.9%)	62 (46.3%)	29 (39.7%)			
≥55	42 (56.8%)	38 (55.1%)	72 (53.7%)	44 (60.3%)			
Gender					0.096	0.78	0.25
Male	56 (75.7%)	45 (65.2%)	95 (70.9%)	46 (63.0%)			
Female	18 (24.3%)	24 (34.8%)	39 (29.1%)	27 (37.0%)			
Location					0.96	0.44	0.54
Upper stomach	21 (28.4%)	24 (34.8%)	32 (23.9%)	21 (28.8%)			
Middle/Lower stomach	53 (71.6%)	45 (65.2%)	102 (76.1%)	52 (71.2%)			
Differentiation degree					0.38	0.56	0.92
Poor differentiation	52 (70.3%)	50 (72.5%)	102 (76.1%)	56 (76.7%)			
Well differentiation	22 (29.7%)	19 (27.5%)	32 (23.9%)	17 (23.3%)			
Stage					0.003	0.099	0.96
ⅠB-ⅡB	40 (54.1%)	30 (43.5%)	40 (29.9%)	22 (30.1%)			
Ⅲ	34 (45.9%)	39 (56.5%)	94 (70.1%)	51 (69.9%)			
**Survival outcomes**							
Patients with recurrence	52 (70.3%)	34 (49.3%)	86 (64.2%)	22 (30.1%)	< 0.001	0.020	< 0.001
Disease-free survival (DFS)							
Mean, months	33.4	71.3	46.3	95.8	< 0.001	0.063	< 0.001
3-year DFS	40.5%	59.4%	41.8%	71.2%	< 0.001	0.16	< 0.001
5-year DFS	32.4%	52.2%	37.3%	69.9%	< 0.001	0.02	< 0.001
Overall survival (OS)							
Mean, months	48.1	81.6	68.3	113.2	0.001	0.048	0.001
3-year OS	71.6%	71.0%	67.2%	83.6%	0.083	0.074	0.011
5-year OS	55.4%	63.8%	59.7%	80.8%	0.001	0.023	0.002

Abbreviation: D1CT: D1 dissection plus chemotherapy; D1CRT: D1 dissection plus chemoradiotherapy; D2CT: D2 dissection plus chemotherapy; D2CRT: D2 dissection plus chemoradiotherapy; ^1^*P* values are derived from Chi-square test or Fisher's exact test for categorical variables, and *P* values are derived from Log rank test for disease-free survival and Overall survival.

**Table 2:** Baseline characteristics and survival outcomes of study groups after matching^1^

Characteristic	D1CT	D1CRT	D2CT	D2CRT	*P*^2^	*P*^2^	*P*^2^
	(n=50)	(n=50)	(n=50)	(n=50)	D1CT vs. D2CRT	D1CRT vs. D2CRT	D2CT vs. D2CRT
Age (years)					0.55	0.69	0.42
<55	23 (46.0%)	23 (46.0%)	24 (48.0%)	20 (40.0%)			
≥55	27 (54.0%)	27 (54.0%)	26 (52.0%)	30 (60.0%)			
Gender					0.67	0.68	0.83
Male	35 (70.0%)	31 (62.0%)	32 (64.0%)	33 (66.0%)			
Female	15 (30.0%)	19 (38.0%)	18 (36.0%)	17 (34.0%)			
Location					0.51	0.83	0.51
Upper stomach	13 (26.0%)	15 (30.0%)	13 (26.0%)	16 (32.0%)			
Middle/Lower stomach	37 (74.0%)	35 (70.0%)	37 (74.0%)	34 (68.0%)			
Differentiation degree					0.82	1.0	0.82
Poor differentiation	38 (76.0%)	37 (74.0%)	38 (76.0%)	37 (74.0%)			
Well differentiation	12 (24.0%)	13 (26.0%)	12 (24.0%)	13 (26.0%)			
Stage					0.84	0.68	0.84
ⅠB-ⅡB	18 (36.0%)	17 (34.0%)	20 (40.0%)	19 (38.0%)			
Ⅲ	32 (64.0%)	33 (66.0%)	30 (60.0%)	31 (62.0%)			
**Survival outcomes**							
Patients with recurrence	33 (66.0%)	31 (62.0%)	32 (64.0%)	16 (32.0%)	0.001	0.003	0.001
Disease-free survival (DFS)							
Mean, months	35.4	54.4	34.1	95.2	< 0.001	0.006	< 0.001
3-year DFS	42.0%	48.0%	50.0%	70.0%	0.005	0.025	0.041
5-year DFS	38.0%	40.0%	40.0%	68.0%	0.003	0.005	0.005
Overall survival (OS)							
Mean, months	44.8	72.8	59.1	110.7	< 0.001	0.011	0.022
3-year OS	66.0%	62.0%	72.0%	84.0%	0.038	0.013	0.15
5-year OS	46.0%	58.0%	64.0%	80.0%	< 0.001	0.01	0.075

Abbreviation: D1CT: D1 dissection plus chemotherapy; D1CRT: D1 dissection plus chemoradiotherapy; D2CT: D2 dissection plus chemotherapy; D2CRT: D2 dissection plus chemoradiotherapy; ^1^Variables adjusted for matching are age, gender, tumor location, differentiated degree, MLR, and stage. ^2^*P* values are derived from Chi-square test or Fisher's exact test for categorical variables, and *P* values are derived from Log rank test for disease-free survival and Overall survival.

**Table 3:** Baseline characteristics and survival outcomes of SEER validation cohort, 2004-2014.

Characteristic	D1CT	D1CRT	D2CT	D2CRT	*P*^1^	*P*^1^	*P*^1^
	(n=457)	(n=929)	(n=844)	(n=1,338)	D1CT vs. D2CRT	D1CRT vs. D2CRT	D2CT vs. D2CRT
Age (years)					0.039	0.22	0.23
<55	122 (26.7%)	273 (29.4%)	248 (29.4%)	426 (31.8%)			
≥55	335 (73.3%)	656 (70.6%)	596 (70.6%)	912 (68.2%)			
Gender					0.61	0.63	0.71
Male	309 (67.6%)	625 (67.3%)	566 (67.1%)	887 (66.3%)			
Female	148 (32.4%)	304 (32.7%)	278 (32.9%)	451 (33.7%)			
Race					< 0.001	< 0.001	< 0.001
White	309 (67.6%)	596 (64.2%)	554 (65.6%)	772 (57.7%)			
Black	59 (15.1%)	158 (17.0%)	113 (13.4%)	190 (14.2%)			
Other	79 (17.3%)	175 (18.8%)	177 (21.0%)	376 (28.1%)			
Differentiation degree					0.010	0.090	0.77
Poor differentiation	304 (66.5%)	648 (69.8%)	621 (73.6%)	977 (73.0%)			
Well differentiation	153 (33.5%)	281 (30.2%)	223 (26.4%)	261 (27.0%)			
Stage					< 0.001	< 0.001	< 0.001
ⅠB-ⅡB	239 (52.3%)	460 (49.5%)	360 (42.7%)	458 (34.2%)			
Ⅲ	218 (47.7%)	469 (50.5%)	484 (57.3%)	880 (65.8%)			
**Survival outcomes**							
Cancer-specific survival (CSS) progression							
Mean, months	67.8	76.0	73.9	82.4	< 0.001	0.012	0.002
3-year CSS	49.6%	54.5%	56.6%	59.9%	< 0.001	0.009	0.12
5-year CSS	44.6%	47.5%	49.8%	54.0%	< 0.001	0.002	0.052
Overall survival (OS)							
Mean, months	60.6	67.5	67.8	76.5	< 0.001	0.001	0.002
3-year OS	45.2%	49.7%	53.3%	56.9%	< 0.001	0.001	0.066
5-year OS	39.6%	40.7%	46.0%	49.8%	< 0.001	< 0.001	0.058

Abbreviation: SEER: Surveillance, Epidemiology, and End Results program; D1CT: D1 dissection plus chemotherapy; D1CRT: D1 dissection plus chemoradiotherapy; D2CT: D2 dissection plus chemotherapy; D2CRT: D2 dissection plus chemoradiotherapy; ^1^*P* values are derived from Chi-square test or Fisher's exact test for categorical variables, and *P* values are derived from Log rank test for cancer-specific survival and Overall survival.

**Table 4:** Multivariate analysis of prognostic factors affecting OS and PFS of study group before and after matching.

Variables	N	Before matching					N	After matching^1^				
		Overall survival			Disease-free survival			Overall survival			Disease-free survival	
		HR (95%CI)^2^	*P*^2^		HR (95%CI)^2^	*P*^2^		HR (95%CI)^2^	*P*^2^		HR (95%CI)^2^	*P*^2^
Age (<55 years as ref.)												
≥55 years	196	0.87 (0.62-1.2)	0.43		0.87 (0.66-1.2)	0.35	110	0.67 (0.43-1.1)	0.085		0.70 (0.48-1.0)	0.065
MLR (<0.32 as ref.)^ 3^												
≥0.32	196	1.5 (1.0-2.3)	0.038		1.63 (1.2-2.2)	0.003	113	1.5 (0.92-2.4)	0.10		1.7 (1.1-2.5)	0.015
Stage (IB-IIB as ref.)												
III	218	1.3 (0.85-1.9)	0.25		1.4 (0.98-1.9)	0.080	126	1.1 (0.72-1.8)	0.57		1.3 (0.89-2.0)	0.18
Treatment (D2CRT as ref.)												
D1CT	69	2.9 (1.5-5.5)	0.001		2.8 (1.7-4.8)	< 0.001	50	3.3 (1.6-6.9)	0.001		2.7 (1.5-4.9)	<0.001
D1CRT	134	1.7 (0.89-3.3)	0.11		1.5 (0.87-2.6)	0.14	50	2.4 (1.1-5.1)	0.023		2.1 (1.1-3.9)	0.018
D2CT	73	2.5 (1.4-4.6)	0.002		2.8 (1.8-4.5)	< 0.001	50	2.3 (1.1-5.0)	0.029		2.9 (1.6-5.3)	0.001

Abbreviation: D1CT: D1 dissection plus chemotherapy; D1CRT: D1 dissection plus chemoradiotherapy; D2CT: D2 dissection plus chemotherapy; D2CRT: D2 dissection plus chemoradiotherapy; HR: hazard ratio; MLR: metastatic lymph node ratio; CI: confidence interval. ^1^Variables adjusted for matching are age, gender, tumor location, differentiated degree, MLR, and stage. ^2^Adjusted variables in the final model are age, MLR, stage, and treatment, hazard ratio and *P* values are derived from Cox proportional hazardous model and Log rank test. ^3^MLR: Metastatic lymph node ration, defined as the ratio of positive lymph nodes in harvested lymph nodes.

## Abbreviations

CI: confidence interval
D1CT: D1 dissection plus adjuvant chemotherapy
D1CRT: D1 dissection plus adjuvant chemoradiotherapy
D2CT: D2 dissection plus adjuvant chemotherapy
D2CRT: D2 dissection plus adjuvant chemoradiotherapy
DFS: disease-free survival
HR: hazard ratio
LAGC: locally advanced gastric adenocarcinoma
MLR: metastatic lymph node ratio
OS: overall survival
PSM: propensity score matching
CT: computed tomography
MRI: magnetic resonance imaging
RCT: randomized controlled trial
