# Supplementary material for: Comparison of Treatment Modalities for Locally Advanced Gastric Cancer: A Propensity Score Matching Analysis
Source: J Cancer. 2020 May 18;11(15):4421–30. doi: 10.7150/jca.41082 (PMC7255382; doi:10.7150/jca.41082)
Supplement: Supplementary file 1 — Supplementary figures and tables. [file jcav11p4421s1.pdf]

Table S1. Treatment Profile for study groups

Regimens ,Cycles	D1CT (n=74)	D1CRT (n=69)	D2CT (n=134)	D2CRT (n=73)
FOLFOX, 4cycles	14	19	20	13
FOLFOX, 6cycles	14	17	37	27
mFOLFOX, 4cycles	17	12	34	14
mFOLFOX, 6cycles	15	15	10	2
Capecitabine	3	0	1	1
Tegafur	3	0	4	4
XELOX, 4cycles	0	4	1	1
SOX, 6cycles	0	0	5	1
EOX	0	0	3	0
EOF	0	0	1	0
5-FU+CF	1	0	0	0
5-FU+L-OHP	2	1	8	1
5-FU+Paclitaxel	1	0	0	1
5-FU+Mitomycin	1	0	0	0
DP	0	1	4	8
Cyclophosphamide+Methotrexate+5-FU	2	0	0	0
Cyclophosphamide+Epirubicin+5-FU	0	0	2	0
Epirubicin+cisplatin+Mitomycin	1	0	1	0
Etoposide+CF	0	0	3	0

Table S2. Multivariate analysis of prognostic factors affecting OS and PFS of SEER validation group.

Variable	Overall survival (OS)			Cancer-specific survival (CSS)		
	n	HR (95% CI)*	*P**	n	HR (95% CI)*	*P**
Age (years)						
< 55 (as reference)	1069	1.0		1069	1	
≥ 55	2499	1.33 (1.21-1.47)	< 0.001	2499	1.24 (1.12-1.37)	< 0.001
Race						
White (as reference)	2231	1.0		2231	1.0	
Black	530	1.01 (0.89-1.13)	0.97	530	0.95 (0.83-1.08)	0.44
Other	807	0.73 (0.66-0.82)	< 0.001	807	0.74 (0.66-0.83)	< 0.001
MLR†						
MLR < 0.32 (as reference)	2186	1.0		2186	1.00	
0.32 ≤ MLR < 1	1382	1.90 (1.71-2.11)	< 0.001	1382	1.91 (1.71-2.12)	< 0.001
Stage						
IB - IIB (as reference)	1517	1.0		1517	1.00	
III	2051	1.88 (1.68-2.01)	< 0.001	2051	2.06 (1.83-2.33)	< 0.001
Treatment						
D2CRT (as reference)	1338	1.0		1338	1.00	
D1CT	457	1.57 (1.37-1.80)	< 0.001	457	1.59 (1.38-1.85)	< 0.001
D1CRT	929	1.20 (1.08-1.34)	0.001	929	1.18 (1.05-1.32)	0.007
D2CT	844	1.31 (1.17-1.47)	< 0.001	844	1.34 (1.18-1.51)	< 0.001

Abbreviation: SEER: Surveillance, Epidemiology, and End Results program; D1CT: D1 dissection plus chemotherapy; D1CRT: D1 dissection plus chemoradiotherapy; D2CT: D2 dissection plus chemotherapy; D2CRT: D2 dissection plus chemoradiotherapy; HR: hazard ratio; MLR: metastatic lymph node ratio; CI: confidence interval.

* Adjusted variables in the final model are age, race, stage, MLR, and treatment, hazard ratio and *P* values are derived from Cox proportional hazardous model and Log rank test.

†MLR: Metastatic lymph node ration, defined as the ratio of positive lymph nodes in harvested lymph nodes.

Table S3. Adverse effect among study groups before and after matching

Adverse effect	D1CT n (%)		D1CRT n (%)		D2CT n (%)		D2CRT n (%)	
	Grade 1/2	Grade 3/4	Grade 1/2	Grade 3/4	Grade 1/2	Grade 3/4	Grade 1/2	Grade 3/4
Before Matching								
Hematologic	15 (20.3)	1 (1.4)	25 (36.2)	2 (2.9)	35 (26.1)	3 (2.2)	8 (11.0)	5 (6.8)
Gastrointestinal	12 (16.2)	5 (6.8)	22 (31.9)	6 (8.7)	19 (14.2)	9 (6.7)	6 (8.2)	8 (11)
Liver toxicity	0 (0)	0 (0)	0 (0)	0 (0)	3 (2.2)	0 (0)	1 (1.4)	0 (0)
Other toxicity	0 (0)	0 (0)	0 (0)	0 (0)	0 (0)	0 (0)	0 (0)	0 (0)
Any adverse event	27 (36.5)	8 (8.2)	47 (68.1)*	8 (8.7)	57 (42.5)	12 (8.9)	15 (20.6)	13 (17.8)
After Matching								
Hematologic	9 (18.0)	1 (2.0)	11 (22.0)	2 (4.0)	10 (20.0)	0 (0)	7 (14.0)	2 (4.0)
Gastrointestinal	8 (16.0)	3 (6.0)	12 (24.0)	4 (8.0)	6 (12.0)	2 (4.0)	4 (8.0)	6 (12.0)
Liver toxicity	0 (0)	0 (0)	0 (0)	0 (0)	0 (0)	0 (0)	1 (2.0)	0 (0)
Other toxicity	0 (0)	0 (0)	1 (2.0)	0 (0)	0 (0)	0 (0)	0 (0)	0 (0)
Any adverse event	17 (34.0)	4 (8.0)	24 (48.0)*	6 (12.0)	16 (32.0)	2 (4.0)	12 (24.0)	8 (16.0)

Abbreviation: D1CT: D1 dissection plus chemotherapy; D1CRT: D1 dissection plus chemoradiotherapy; D2CT: D2 dissection plus chemotherapy; D2CRT: D2 dissection plus chemoradiotherapy.

*$P < 0.05$ for select group compared with D2CRT group.

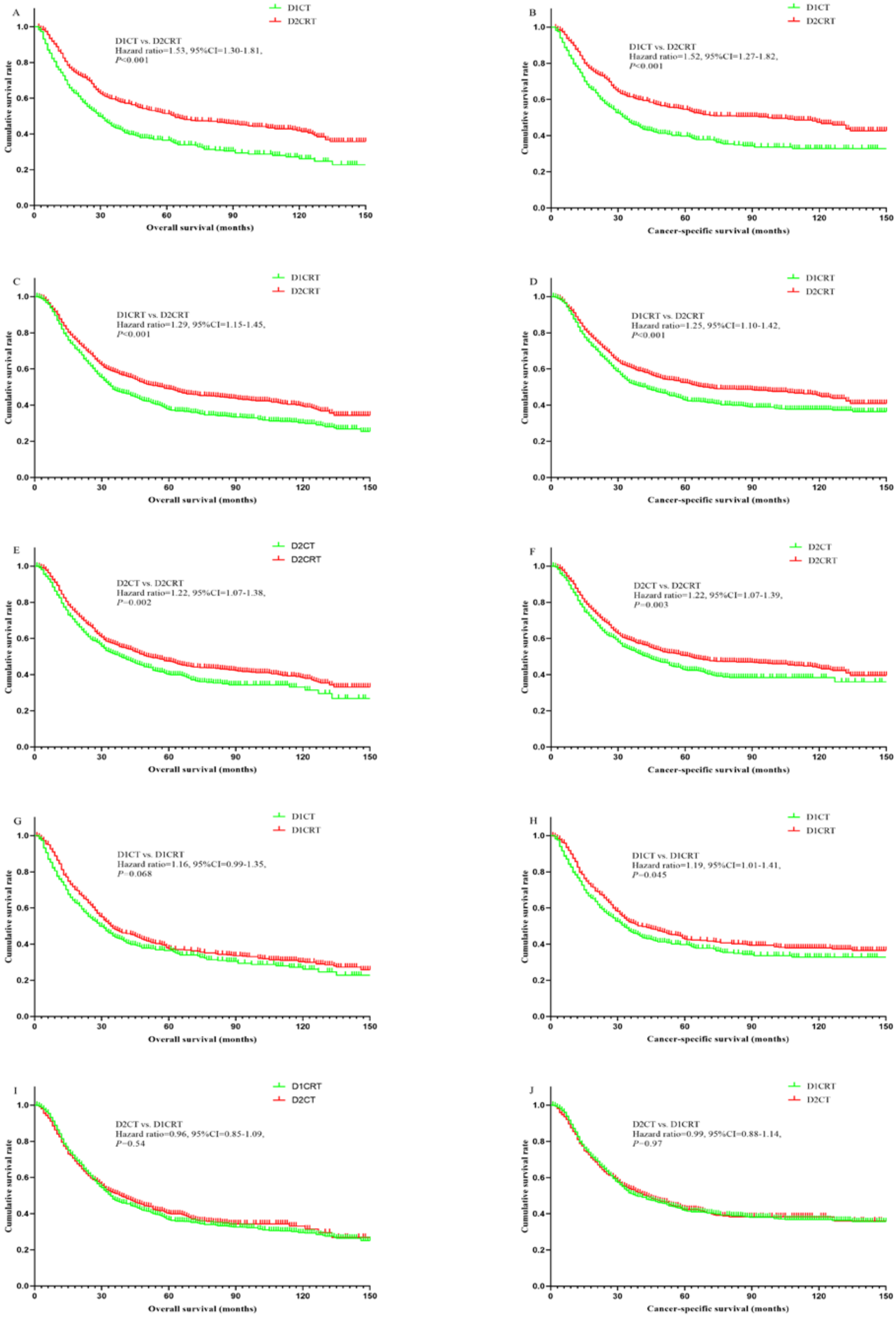

Figure S1. Kaplan-Meier curves of overall survival (OS) and cancer-specific survival (CSS) relative to each group of SEER validation cohort after matching.

Note: The Kaplan-Meier survival curves of OS (A) and CSS (B) for D1CT and D2CRT groups; the Kaplan-Meier survival curves of OS (C) and CSS (D) for D1CRT and D2CRT groups; the Kaplan-Meier survival curves of OS (E) and CSS (F) for D2CT and D2CRT groups; the Kaplan-Meier survival curves of OS (G) and CSS (H) for D1CT and D1CRT groups; the Kaplan-Meier survival curves of OS (I) and CSS (J) for D1CT and D2CRT groups

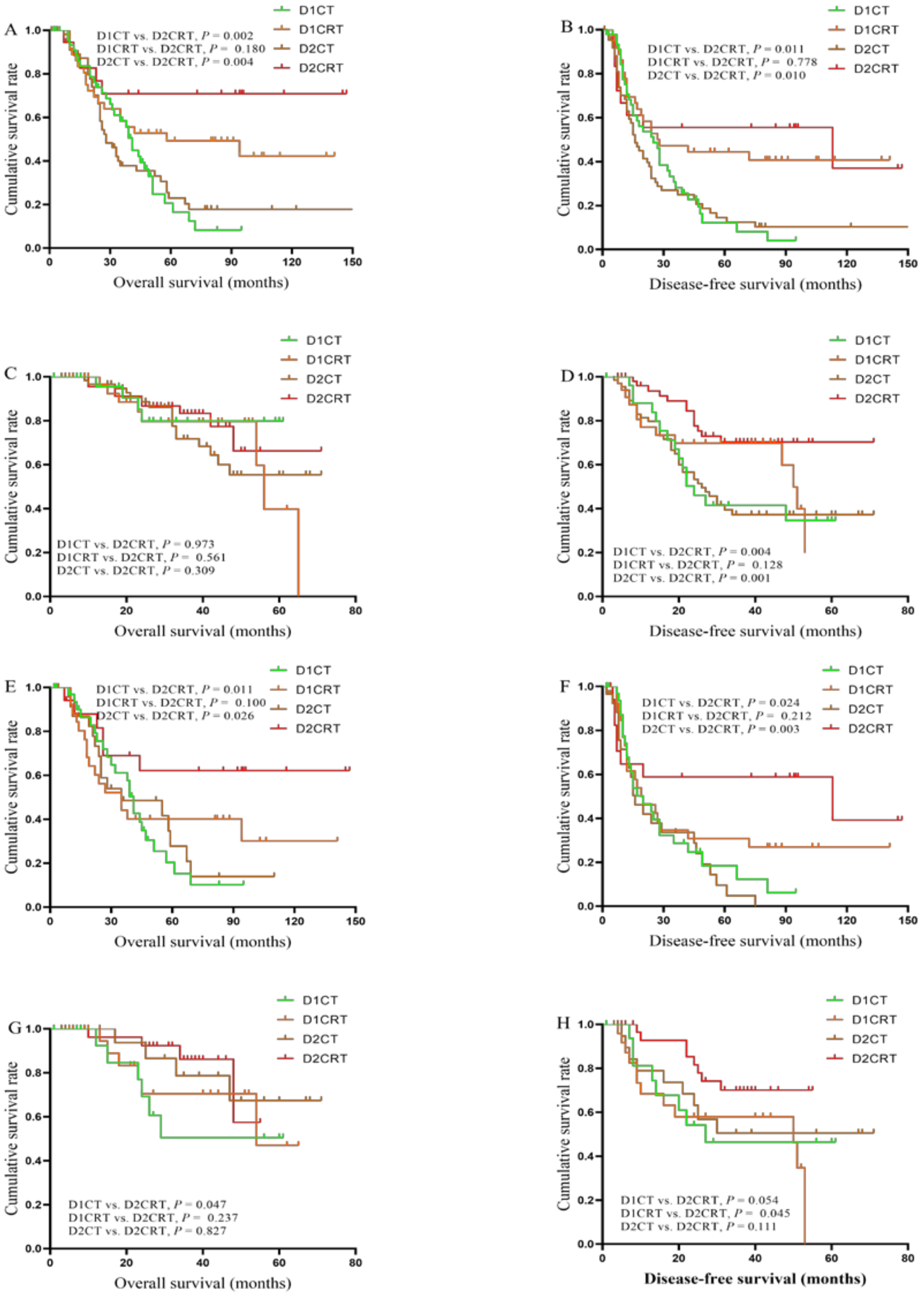

Figure S2. Kaplan-Meier curves of overall survival (OS) and disease-free survival (DFS) relative to each group both before (A-D) and after matching (E-H).

Note: The OS and DFS curves of patients received surgery before 2011 were shown in Figure A-B (before matching) and Figure E-F (after matching); those received surgery after 2011 showed in Figure C-D (before matching) and Figure G-H (after matching).
